# Partial functional conservation of IRX10 homologs in *physcomitrella patens* and *Arabidopsis thaliana* indicates an evolutionary step contributing to vascular formation in land plants

**DOI:** 10.1186/1471-2229-13-3

**Published:** 2013-01-03

**Authors:** Emma Hörnblad, Mikael Ulfstedt, Hans Ronne, Alan Marchant

**Affiliations:** 1 UPSC, Department of Forest Genetics and Plant Physiology, Swedish University of Agricultural Sciences, Umeå, SE-90183, Sweden; 2 Department of Microbiology, Uppsala Biocenter, Swedish University of Agricultural Sciences, Box 7025, Uppsala, SE-750 07, Sweden; 3 Centre for Biological Sciences, Life Sciences Building 85, University of Southampton, Southampton, SO17 1BJ, UK

**Keywords:** Plant cell wall, Hemicellulose, *Arabidopsis thaliana*, *Physcomitrella patens*, Glycosyltransferases, Xylan

## Abstract

**Background:**

Plant cell walls are complex multicomponent structures that have evolved to fulfil an essential function in providing strength and protection to cells. Hemicelluloses constitute a key component of the cell wall and recently a number of the genes thought to encode the enzymes required for its synthesis have been identified in *Arabidopsis*. The acquisition of hemicellulose synthesis capability is hypothesised to have been an important step in the evolution of higher plants.

**Results:**

Analysis of the *Physcomitrella patens* genome has revealed the presence of homologs for all of the *Arabidopsis* glycosyltransferases including IRX9, IRX10 and IRX14 required for the synthesis of the glucuronoxylan backbone. The *Physcomitrella* IRX10 homolog is expressed in a variety of moss tissues which were newly formed or undergoing expansion. There is a high degree of sequence conservation between the *Physcomitrella* IRX10 and *Arabidopsis* IRX10 and IRX10-L. Despite this sequence similarity, the *Physcomitrella IRX10* gene is only able to partially rescue the *Arabidopsis irx10 irx10-L* double mutant indicating that there has been a neo- or sub-functionalisation during the evolution of higher plants. Analysis of the monosaccharide composition of stems from the partially rescued *Arabidopsis* plants does not show any significant change in xylose content compared to the *irx10 irx10-L* double mutant. Likewise, knockout mutants of the *Physcomitrella IRX10* gene do not result in any visible phenotype and there is no significant change in monosaccharide composition of the cell walls.

**Conclusions:**

The fact that the *Physcomitrella* IRX10 (PpGT47A) protein can partially complement an *Arabidopsis irx10 irx10-L* double mutant suggests that it shares some function with the *Arabidopsis* proteins, but the lack of a phenotype in knockout lines shows that the function is not required for growth or development under normal conditions in *Physcomitrella*. In contrast, the *Arabidopsis irx10* and *irx10 irx10-L* mutants have strong phenotypes indicating an important function in growth and development. We conclude that the evolution of vascular plants has been associated with a significant change or adaptation in the function of the *IRX10* gene family.

## Background

The components and structures of plant cell walls have evolved over millions of years, resulting in a diverse range of traits and functions. From a comparison of cell walls of land plants, it is apparent that there are some common structural components, and that at least a proportion of the cell wall biosynthesis machinery is likely to be conserved among embryophytes. Studies have shown that members of the charophycean green algae, the closest relatives of land plants, possess cell walls that share many basic components with the embryophyte primary cell wall including a number of polysaccharides, structural proteins and lignin [1-3]. Although some of the components of the plant cell wall may have formed via convergent evolution, it seems likely that others were present in a common ancestor and were retained following colonisation of the land by plants. It can be hypothesised that glycosyltransferases (GTs) required for synthesis of some of the cell wall polysaccharides will be conserved between lower and higher plants.

Comparative biochemical analyses based on enzymatic digestion, linkage analyses and immunolocalisation of polysaccharide epitopes have been used to show similarities and differences between the cell wall components of angiosperms and bryophytes [1,4-9]. Some bryophyte cell wall polymers including cellulose, glucuronoxylan and rhamnogalacturonan II have been isolated and characterized [4,10-12], but very little is known about the evolutionary relationship between the genes encoding the enzymes required for cell wall biosynthesis in lower and higher plants. The cell wall organisation of *Physcomitrella patens* is similar to the angiosperm primary cell wall and almost all major non-lignin cell wall components including cellulose, hemicelluloses (except xylogalacturonan), pectins and arabinogalactan proteins (AGPs) found in angiosperms are also present in bryophytes though in different proportions. There are however some components such as mixed-linkage β-glucans which are found in some land plants but not in the bryophytes.

Cell walls of higher plants have significantly more xylan than lower plants such as the bryophytes indicating that a change in the proportion of the hemicellulose polymer present in cell walls may have been a key step in plant evolution [6,9,12,13]. The hemicellulose glucuronoxylan (GX) is the second most abundant polymer in hardwood secondary cell walls, and is a major component of dicot wood [14,15]. There is a high degree of conservation of both the GX biosynthesis machinery and the structure of GX between *Arabidopsis* and *Populus*[16-22], but it is only recently that equivalent information has been obtained from lower plants including bryophytes [12].

The structure of GX in angiosperm secondary cell walls is highly conserved consisting of a partly acetylated β-D-(1 → 4)-Xyl*p* backbone substituted with either 4-O-methylated (Me)- or non-methylated glucuronic acid (Glc*p*A) side chains [23]. Analysis of GX from spruce, birch, aspen and *Arabidopsis* has revealed a conserved oligosaccharide at the reducing end comprised of β-D-Xyl*p*-(1 → 3)-α-L-Rha*p*-(1 → 2)-α-D-Gal*p*A-(1 → 4)-D-Xyl [14,23-28]. In the moss *Physcomitrella patens*, a xylan polymer substituted at the O-2 position by α-D-Glc*p*A has been detected. However, in contrast to higher plants the moss xylan was not substituted by 4-O-Me GlcpA and the presence of a reducing end oligosaccharide was also not detected [12]. Twelve glycosyltransferase genes have been identified to date in *Arabidopsis* that are involved in GX synthesis; namely *IRREGULAR XYLEM 10* (*AtIRX10*), *AtIRX10-Like* (*AtIRX10-L*), *AtIRX9*, *AtIRX9-L*, *AtIRX14*, *AtIRX14-L*, *AtFRAGILE FIBER 8* (*AtFRA8*), *AtFRAGILE FIBER 8 HOMOLOG* (*AtF8H*), *AtIRX8*, *AtPARVUS*, *AtGUX1* and *AtGUX2*[19,29-39]. Although enzymatic activity has yet to be demonstrated for any of the putative GX synthesis enzymes, microsome experiments support the hypothesis that AtIRX9, AtIRX10 and AtIRX14 are involved in synthesis of the β-D-(1 → 4)-xylan backbone [33,40,41] while the AtIRX8, AtPARVUS and AtFRA8 enzymes are instead thought to function to synthesise the reducing end oligosaccharide [19,31,32]. Evidence for conservation of the GX biosynthetic machinery between herbaceous and woody plants has come from the finding that *Populus* homologs of putative *Arabidopsis* GX synthesis enzymes are able to complement the corresponding *Arabidopsis* mutants [16-18,22,42]. Two *Populus* IRX10 homologs have been shown to be functionally conserved with the *Arabidopsis* IRX10 homolog as they can fully restore the *Arabidopsis irx10 irx10-L* double mutant to a wild-type phenotype (Hörnblad & Marchant, unpublished data).

In this study, *Physcomitrella* has been used to investigate the origin and functional role of the GT47 or *IRX10* gene family. Results obtained suggest a common ancestor for the *IRX10* gene family in *Populus*, *Arabidopsis* and *Physcomitrella*, and supports a partial conservation of the components in the GX biosynthetic machinery. In *Arabidopsis* and *Populus,* expression of a number of *IRX10* gene family members is strongly correlated with the vasculature whereas expression in *Physcomitrella* is found in tissues undergoing expansion rather than in water-conducting cells. Furthermore, the lack of increased xylose content in the *Arabidopsis irx10 irx10-L* double knockout plants complemented with *PpGT47A* reveal that although the proteins are likely to share a common genetic origin, they are no longer directly functionally interchangeable.

## Results

### The *Physcomitrella* genome contains genes encoding homologs of all known enzymes involved in synthesis of the glucuronoxylan backbone

**Figure 1:**
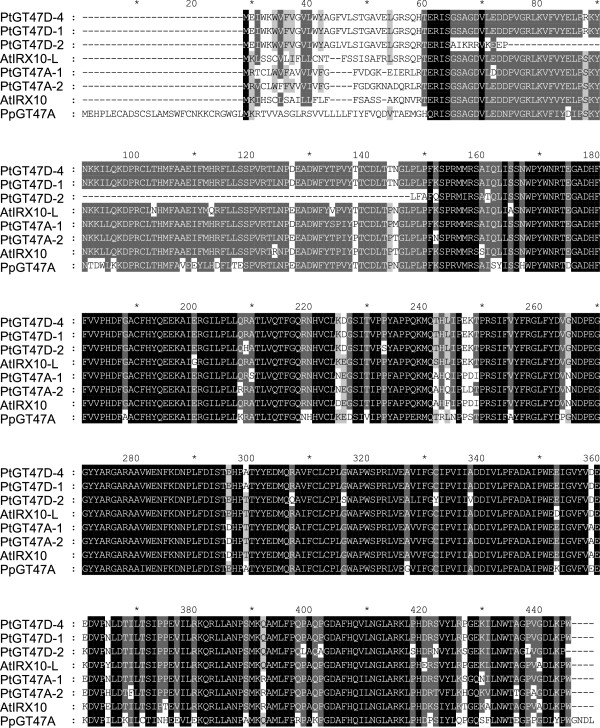
**Sequence alignment of the *Arabidopsis*, *Populus *and *Physcomitrella *IRX10 protein family.** IRX10 related protein sequences from *Arabidopsis*, *Populus* and *Physcomitrella* were aligned using Clustal-X. Shading indicates 100% conservation (black), 80% conservation (dark grey) or 60% conservation (light grey).

**Figure 2:**
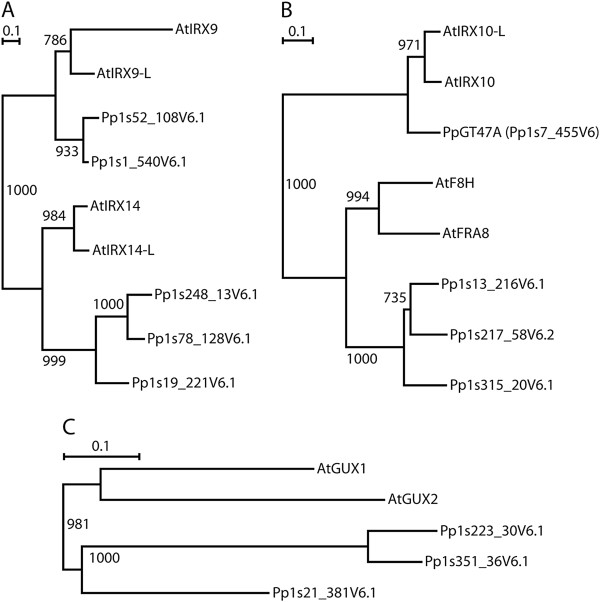
**Phylogenetic trees for GX related proteins in *Arabidopsis *and *Physcomitrella.*** Predicted protein sequences were aligned within each family, and a tree was computed using the neighbour-joining method [46] with gapped positions being excluded and branch length corrections for multiple substitutions enabled. The bars represent a PAM value (percent accepted mutations) of 10%. The numbers shown at the branch points are bootstrap values derived from 1000 randomized sequences. **A**) GT43 family. **B**) GT47 family. **C**) GT8 family.

Work carried out in *Arabidopsis* has shown that there are multiple enzymes involved in the synthesis of the glucuronoxylan backbone (IRX9, IRX10 and IRX14), substitution of the backbone (GUX1/GUX2) and synthesis of the reducing end oligosaccharide (IRX8, FRA8 and PARVUS) [30,31,40]. To determine whether there is conservation of the genes required for xylan biosynthesis between higher and lower plants, each of the *Arabidopsis* proteins proposed to function in the synthesis of glucuronoxylan were used to BLAST search the *Physcomitrella* genome. A single putative IRX10 homolog is present in *Physcomitrella* encoded by Pp1s7_455V6 (*PpGT47A*) (Table 1) which shows a high degree of conservation with the *Arabidopsis* and *Populus trichocarpa* IRX10 proteins (Figure 1). The N-terminal regions of the *Arabidopsis* and *Populus* IRX10 proteins are the most divergent domains and contain predicted signal peptides in AtIRX10, AtIRX10-L, PtGT47A-1 and PtGT47A-2 whereas the N-terminal region of PpGT47A has a predicted transmembrane domain in common with two of the other *Populus* homologs PtGT47D-1 and PtGT47D-4 [43,44] (Table 2). Two putative homologs were found for AtIRX9/IRX9-L (Pp1s52_108V6.1 and Pp1s1_540V6.1), whereas three putative homologs were each identified for AtIRX14/IRX14-L (Pp1s248_13V6.1, Pp1s78_128V6.1 and Pp1s19_221V6.1), AtFRA8/F8H (Pp1s13_216V6.1, Pp1s217_58V6.2 and Pp1s315_20V6.1), and AtGUX1/GUX2 (Pp1s21_381V6.1, Pp1s351_36V6.1 and Pp1s223_30V6.1) (Figure 2). Interestingly, there were no closely related sequences found for AtIRX8. Although several sequences were identified that exhibited a 60% similarity with PARVUS, all of them exhibited a higher degree of similarity with other *Arabidopsis* proteins and so were not considered likely to be homologs of PARVUS (Table 1). The degree of similarity between the *Arabidopsis* and *Physcomitrella* homologs was between 25% and 48% except in the case of the PpGT47A which exhibited 76% similarity with AtIRX10 and AtIRX10-L. All genes are present in two or three copies encoding closely related proteins in both species, with the exception of *PpGT47A* which is unique (Figure 2). In all cases, duplicated or triplicated genes within a species encode proteins that cluster together (Figure 2). This suggests that gene duplications have occurred in both species after they split from each other. However, concerted evolution of related genes cannot be ruled out as a reason for close similarity, at least not in *Physcomitrella*, where it has been previously observed [45]. The high degree of sequence similarity exhibited between the *Arabidopsis* IRX10 and the *Physcomitrella* GT47A proteins further suggests that they may exhibit functional conservation and thus may give clues as to the evolutionary origin of the GX biosynthesis machinery in higher plants.

**Table 1:** **Similarity between the *Arabidopsis *GX synthesis proteins and their respective *Arabidopsis *and *Physcomitrella *homologs**

***Arabidopsis *protein**	**vs. gene model**	**ClustalW identity score(%)**
**AtIRX10-L (At5g61840)**	*AtIRX10 (At1g27440)*	86.17
	*PpGT47A (Pp1s7_455V6)*	75.75
**AtIRX10 (At1g27440)**	*AtIRX10-L (At5g61840)*	86.17
	*PpGT47A (Pp1s7_455V6)*	76.39
**AtFRA8 (At2g28110)**	*AtF8H (At5g22940)*	57.81
	*Pp1s315_20V6.1*	45.31
	*Pp1s217_58V6.1*	48.33
	*Pp1s13_216V6.1*	45.98
**AtF8H (At5g22940)**	*AtFRA8 (At2g28110)*	57.81
	*Pp1s315_20V6.1*	43.07
	*Pp1s217_58V6.1*	47.86
	*Pp1s13_216V6.1*	43.92
**AtIRX9 (At2g37090)**	*AtIRX9-L (At1g27600)*	29.06
	*Pp1s52_108V6.1*	29.06
	*Pp1s1_540V6.1*	27.92
**AtIRX9-L (At1g27600)**	*AtIRX9 (At2g37090)*	29.06
	*Pp1s52_108V6.1*	44.92
	*Pp1s1_540V6.1*	44.16
**AtIRX14 (At4g36890)**	*AtIRX14-L (At5g67230)*	61.79
	*Pp1s248_13V6.1*	29.33
	*Pp1s19_221V6.1*	27.74
	*Pp1s78_128V6.1*	30.46
**AtIRX14-L (At5g67230)**	*AtIRX14 (At4g36890)*	61.79
	*Pp1s248_13V6.1*	24.80
	*Pp1s19_221V6.1*	26.83
	*Pp1s78_128V6.1*	28.17
**AtGUX1 (At3g18660)**	*AtGUX2 (At4g33330)*	39.60
	*Pp1s21_381V6.1*	44.75
	*Pp1s351_36V6.1*	34.05
	*Pp1s223_30V6.1*	34.35
**AtGUX2 (At4g33330)**	*AtGUX1 (At3g18660)*	39.60
	*Pp1s21_381V6.1*	42.93
	*Pp1s351_36V6.1*	34.23
	*Pp1s223_30V6.1*	33.05
**AtIRX8**	*No homolog found*	
**AtPARVUS**	No obvious homolog found*	

Gene models were considered putative homologs if the *Arabidopsis* protein that was used for the Blast was the *Arabidopsis* protein which exhibited most similarity. *Although three *Physcomitrella* gene models were identified with the corresponding protein exhibiting between 49.57 and 51.57% similarity to PARVUS, the sequences all exhibited stronger homology to at least 10 other *Arabidopsis* proteins, and were therefore not considered as obvious homologs. (ClustalW).

**Table 2:** **Phobius transmembrane and signal sequence prediction for members of the *Arabidopsis*, *Populus *and *Physcomitrella *IRX10 family of proteins**

**Protein**	**Amino acid**	**Prediction**	**Protein**	**Amino acid**	**Prediction**
**AtIRX10**	1-21	Signal	**AtIRX10-L**	1-19	Signal
	1-5	N-region		1-2	N-region
	6-16	H-region		3-13	H-region
	17-21	C-region		14-21	C-region
	22-412	Non-cytoplasmic		22-415	Non-cytoplasmic
**PtGT47A-1**	1-19	Signal	**PtGT47A-2**	1-19	Signal
	1-2	N-region		1-2	N-region
	3-14	H-region		3-14	H-region
	15-19	C-region		15-19	C-region
	20-412	Non-cytoplasmic		20-413	Non-cytoplasmic
**PtGT47D-1 and PtGT47D-4**	1-5	Cytoplasmic	**PpGT47A**	1-31	Cytoplasmic
	6-26	Transmembrane		32-51	Transmembrane
	27-417	Cytoplasmic		52-449	Non-cytoplasmic

IRX10 related protein sequences from *Arabidopsis, Populus* and *Physcomitrella* were analysed using the Phobius programme (http://www.phobius.sbc.su.se) for the presence of N-terminal signal sequences and the locations of the hydrophobic predicted transmembrane core (H-region) flanked by the N- and C- regions.

### The *PpGT47A* expression pattern indicates a role during early stages of development in *physcomitrella*

**Figure 3:**
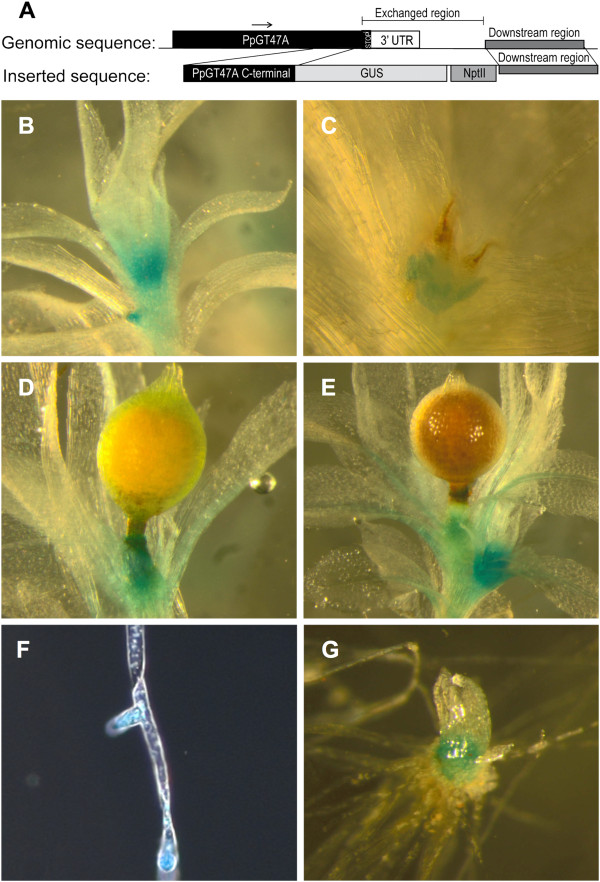
**The expression pattern of *PpGT47A *in *Physcomitrella*, analyzed by histochemical staining of stable *pGT47A:GUS *lines.** (**A**) Schematic drawing describing the GUS construct inserted into the *Physcomitrella* genome. (**B**) Adult gametophore shows GUS staining in the apical region and in the emerging gametophore on the side of the adult gametophore. (**C**) GUS staining of the antheridia. (**D**) GUS staining in tissue below the immature sporophyte. (**E**) Expression peak in young gametophore growing on the side of old gametophore. (**F**) Protonema side branches undergoing tip growth exhibit peaks in GUS expression. (**G**) Bud/juvenile gametophore exhibiting GUS staining.

To investigate whether expression of *PpGT47A* is associated with specific tissues or developmental stages of growth, the *uidA* reporter gene was fused to the 3^′^ end of the *PpGT47A* gene. The construct (Figure 3A) was introduced into the *Physcomitrella* genome at the endogenous *PpGT47A* site via homologous recombination. The pattern of pGT47A expression in *Physcomitrella* was analysed by histochemical staining of 4 independent stable pGT47A-GUS lines, each of which showed a similar staining pattern. Expression was localised predominantly to tissues which were newly formed or undergoing expansion. Strong expression was observed in the apical region of the adult gametophore (Figure 3B), new branches forming on the side of the mature gametophore (Figure 3E) and in the tissue immediately basal to the immature sporophyte (Figure 3D). Staining was also observed in the antheridia (Figure 3C). A peak of expression was seen at the tips of the protonema side branches (Figure 3F) and the lateral buds of the chloronema (Figure 3G).

### The *PpGT47A* gene partially complements the *Arabidopsis irx10 irx10-L* double mutant

**Figure 4:**
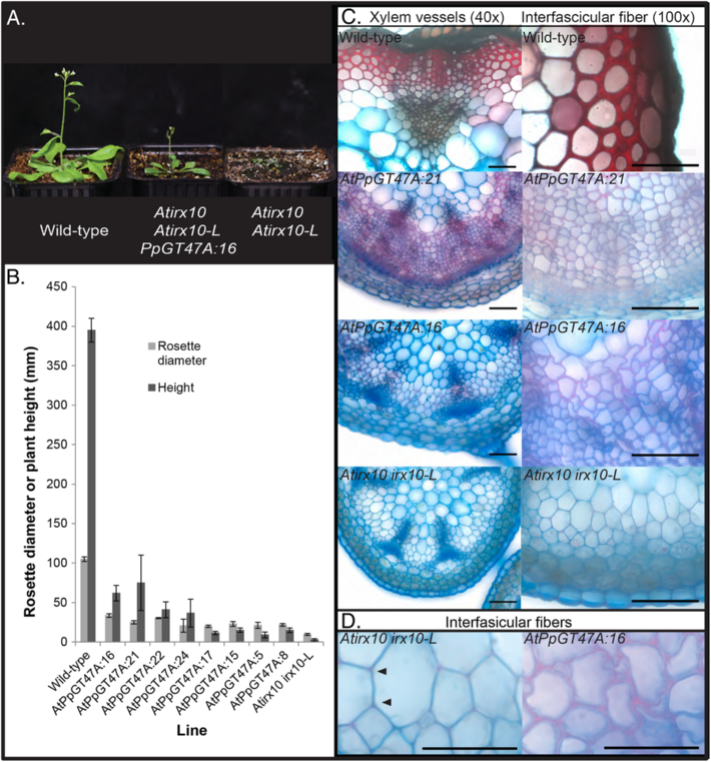
**The *Physcomitrella IRX10 *homolog expressed in the *Arabidopsis irx10 irx10-L *double mutant. A**) *Arabidopsis irx10 irx10-L* double mutant partially complemented by p35S:*PpGT47A*. **B**) Rosette diameter and inflorescence height of *PpGT47A* complemented *Arabidopsis* plants. Data obtained from between 2 and 7 biological repeats per line. Each line represents one independent transformation event. **C**) Sections from 8-week old basal stem tissue of Columbia wild-type, *Arabidopsis irx10 irx10-L* double mutant complemented with the *Physcomitrella IRX10* homolog, and the *Arabidopsis irx10 irx10-L* double mutant. **D**) Higher magnification of the safranin stained tissues shown in C. Safranin staining in the cell corners of the *irx10 irx10-L* double mutant is indicated by arrowheads. Scale bar 50 μm (**C**) or 20 μm (**D**).

To establish whether the function of the PpGT47A enzyme is conserved between *Arabidopsis* and *Physcomitrella*, the *PpGT47A* gene under control of the 35S cauliflower mosaic virus (CaMV) constitutive promoter was expressed in the *Arabidopsis irx10 irx10-L* double mutant background. The *PpGT47A* gene partially rescued the *irx10 irx10-L* phenotype with plants forming a short inflorescence stem without the need for a protective growing cover that is normally required by the double mutant [35] (Figure 4A). Rosette diameters of complemented plants were increased by 2- to 3-fold and the inflorescence stems were between 3- and 28-fold taller compared to the *irx10 irx10-L* plants grown under a plastic cover (Figure 4B). Safranin staining was performed on basal stem sections of the *Arabidopsis* wild-type, *irx10 irx10-L* double mutant and complemented plants to identify regions of lignification. In *Arabidopsis* wild-type plants, the cell walls of the xylem vessels and interfascicular fibers were heavily stained around the whole cell (Figure 4C). In contrast, the *irx10 irx10-L* plants exhibited only a weak signal at the corners of the cell junctions indicating initiation of lignification, with virtually no evidence of secondary cell wall deposition in any other regions of the cell walls (Figure 4D). Irregular and variable safranin staining could be detected throughout the xylem and interfasicular fiber cell walls of stem sections from the complemented plants although the staining was less intense than in the wild-type. Despite the apparent increase in secondary cell wall production, the cell walls of *irx10 irx10-L* plants expressing *PpGT47A* were thinner than wild-type cell walls and displayed a collapsed xylem vessel phenotype (Figure 4C,D).

**Figure 5:**
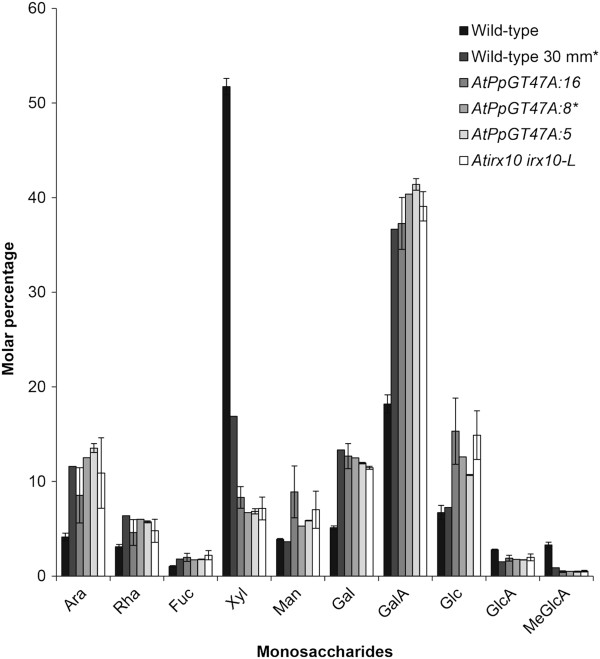
**The *PpGT47A *gene does not rescue the monosaccharide composition of *Arabidopsis irx10 irx10-L *mutant stem tissue.A**) Monosaccharide composition of stems material isolated from mature *Arabidopsis* wild-type plants, young wild-type plants with a 30mm stem, *irx10 irx10-L* double mutants and 3 independent lines of *irx10 irx10-L* double mutants complemented by the *Physcomitrella IRX10* homolog (*AtPpGT47A:16*, *AtPpGT47A:8*, *AtPpGT47A:5*). Data from complemented plants are based on pooled stem material in three technical replicates for each of 3 independent biological samples pooled from multiple plants (except for samples indicated by * where only a single pooled sample was used). Data is displayed as molecular percentage of measured sugars obtained by TMS analysis from 500 μg of amylase treated AIR starting material for each sample. Error bars represent standard deviation based on three replicates.

Monosaccharide analysis was performed on cell wall material isolated from stems of the complemented plants to determine the biochemical basis for the partial complementation phenotype (Figure 5). There were no significant differences in the levels of the monosaccharides measured between the complemented lines and the *irx10 irx10-L* double mutant. In particular, it is noteworthy that the level of xylose remained low in the *PpGT47A* expressing lines in contrast to the results obtained when the *Arabidopsis irx10 irx10-L* double mutant was complemented with the *Arabidopsis IRX10* gene [35].

### *Ppgt47A* Knockout lines show no decrease in xylose or glucuronic acid content compared to wild-type

**Figure 6:**
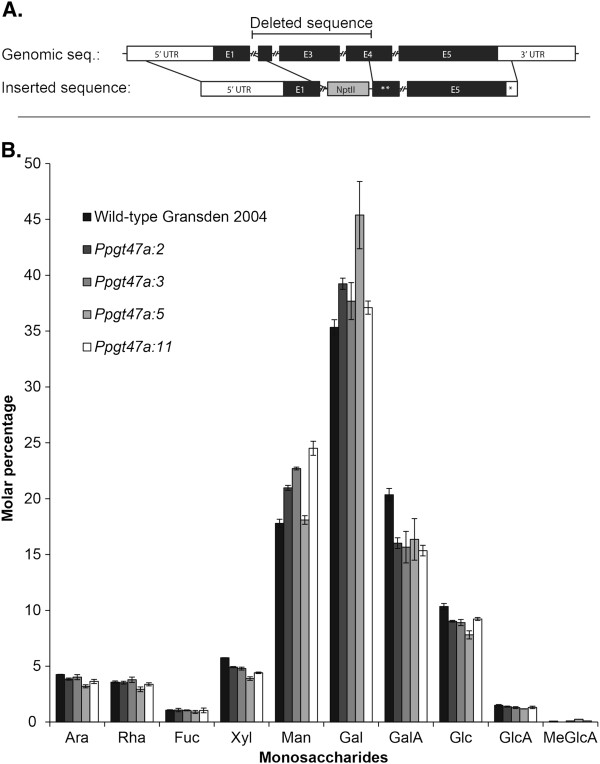
**Knockout of the *Physcomitrella PpGT47A *gene does not cause a significant change in cell wall monosaccharide composition.A**) Schematic drawing of the *PpGT47A* gene and the knock-out construct which was introduced via homologous recombination to obtain knockout mutants in *Physcomitrella*. ** corresponds to a truncated version of the E4 sequence and * corresponds to a truncated version of the 3^′^UTR sequence which are indicated in the upper genomic sequence. **B**) Monosaccharide analysis of gametophore cell walls from *Physcomitrella* wild-type and 4 independent *gt47a* knockout mutants. Data were obtained from 3 technical replicates of two pooled samples for the wild-type and knockout *Ppgt47a:3*, and three pooled samples for knockouts *Ppgt47a:2*; *Ppgt47a:5* and *Ppgt47a:11* .

*Arabidopsis irx10* and *irx10-L* knockout mutants have proven to be informative in elucidating the function of the encoded proteins. In order to further investigate the function of PpGT47A, *Physcomitrella gt47A* knockout mutants were generated. A construct was made in which the *nptII* coding sequence was inserted into the *PpGT47A* gene deleting part of exons 2 and 4 and the whole of exon 3. The construct was introduced into the *Physcomitrella* genome via homologous recombination resulting in the deletion of the central region of the endogenous *PpGT47A* gene to create a likely null knockout mutant. Knockouts were analysed by PCR using primers spanning the recombination site to identify lines in which the endogenous *PpGT47A* gene had been disrupted. The gametophores of the knockout lines were visually similar to wild-type with no obvious morphological changes when grown on BCD media (Additional file 1: Figure S1). Further experiments were carried out to test whether varying the composition of the growth media by addition of different sugars or hormones under different light conditions (see materials and methods for details) revealed any phenotypic changes in the knockout lines but none were observed (data not shown). Monosaccharide analysis of gametophore cell walls from the knockout lines (Figure 6) did not show any significant differences compared to the wild-type. Of particular interest is the observation that there was no difference in xylose content between knockout and wild-type gametophores in contrast to the results obtained for the *Arabidopsis irx10* and *irx10-L* mutants [35].

## Discussion

An important developmental adaptation of land plants involved the ability to form conductive tissues allowing efficient exploitation of available mineral and water resources. It has been suggested that the xylan polymer could have provided a pre-adaptive advantage to ancestors of vascular plants and contributed to the evolution of efficient water conducting systems [47]. Detection of glucuronoxylan in *Physcomitrella*[9,12] shows that the xylan polymer was present in a common ancestor prior to divergence of the bryophytes and vascular plants. The finding that the *Physcomitrella PpGT47A* gene is able to partially substitute for the lack of function of *IRX10* or *IRX10-L* in *Arabidopsis* together with the high degree of sequence conservation further strengthens the argument that there was a common ancestor protein. Despite this, components of the GX synthesis machinery appear to have evolved with the result that the *Arabidopsis* IRX10, IRX10-L and the *Physcomitrella* GT47A proteins although apparently originating from a common ancestor, do not exhibit identical functions or activities.

It has been speculated that the *Arabidopsis* IRX10 protein forms a complex together with AtIRX9 and AtIRX14 and that this complex is responsible for the synthesis of the backbone of GX [39,40]. AtGUX1 and AtGUX2 are proposed to add the GlcA side chains to the backbone [38] while IRX8, FRA8 and PARVUS are thought to synthesise the reducing end oligosaccharide [19,29,31,32,40]. Interestingly, *Physcomitrella* homologs can be identified for all of the known GX synthesis proteins with the exception of IRX8 and PARVUS. Recent studies suggests that xylan structure in *Physcomitrella* differs from that of higher plants and may as in the grasses lack the reducing end oligosaccharide which otherwise is thought to be universally conserved amongst the dicots. The ability to synthesise the reducing end oligosaccharide structure thus may be a relatively recent adaptation that is only found in some higher plants.

A previous study utilised a number of anti-xylan antibodies which recognise distinct epitopes to localise xylan in *Physcomitrella*[12]. These antibodies produced different labelling patterns – for example LM11 which strongly labels axillary hair cells and CCRC-M137 which shows labelling of leaf cell walls in addition to the axillary hair cells. In this study, the Physcomitrella *IRX10* promoter was found to direct expression of the GUS reporter in the apical region of the adult gametophore, new branches forming from the gametophore and tissues basal to the immature sporophyte (Figure 3). There was no preferential staining of axillary hairs observed in any of the GUS lined studied. However, the antibody recognition of xylan in axillary hairs does not rule out presence of xylan epitopes masked by other cell wall components in other *Physcomitrella* cell wall tissues. Furthermore, the possibility that PpIRX10 functions in making a different polysaccharide or makes a form of xylan in *Physcomitrella* which is not recognised by the currently available anti-xylan antibodies cannot be discounted. In the absence of a functional enzymatic assay for PpIRX10 it is currently not possible to be certain about its enzymatic activity, although it is reasonable to hypothesise that it plays a role in xylan biosynthesis, a conclusion which is supported by the partial complementation of the *Arabidopsis irx10 irx10-L* double mutant.

### Failure of *PpGT47A* to fully complement the *irx10 irx10-L Arabidopsis* mutant supports functional divergence between the *Arabidopsis* and *physcomitrella* IRX10 family of proteins

The finding that the *Physcomitrella* GT47A protein only partially rescues the *Atirx10 irx10-L* double mutant demonstrates that although the proteins are highly conserved at the sequence level, there are important differences which influence function. There are a number of possible reasons for these apparent functional differences. Firstly, the enzymatic activity of PpGT47A may be different to that of the *Arabidopsis* IRX10 or IRX10-L proteins. Despite the high degree of sequence conservation between the *Arabidopsis* and *Physcomitrella* IRX10 proteins, subtle amino acid differences could give an altered enzymatic activity but in the absence of a functional enzymatic assay this cannot currently be tested. Alternatively, PpGT47A could have the same enzymatic activity as the *Arabidopsis* IRX10 but the interaction between IRX9 and/or IRX14 to form a putative functional complex may not be optimal. A third possibility is that the targeting or subcellular localisation of PpGT47A is not correct when expressed in *Arabidopsis*. The highly divergent N-terminal domain that in the *Arabidopsis* IRX10 protein encodes a predicted signal peptide, in *Physcomitrella* instead forms a predicted transmembrane domain (Table 2). Thus, mislocalisation and/or a different conformation of the PpGT47A protein compared to AtIRX10 may combine to produce only a small amount of a functional GX synthesis complex or alternatively a complex with low activity in the partially complemented *Arabidopsis* plants.

### Evolutionary adaption of the IRX10 family of enzymes

The *Arabidopsis IRX10* and *Populus PtGT47A-1* genes are expressed within the vascular tissue made up mainly of cells rich in secondary cell walls. Bryophyte water conducting tissues in contrast do not form thickened cell walls and so it is unsurprising that the expression pattern of *PpGT47A* differs from that of the *Arabidopsis* homologs. The ability of *PpGT47A* to partially complement the *Arabidopsis irx10 irx10-L* double mutant plants shows that there is a degree of functional overlap between the *Arabidopsis* and *Physcomitrella* enzymes but that either activity, conformation or targeting of the enzymes prevent full rescue of the wild-type phenotype. Interestingly, the degree of similarity is higher between PpGT47A and AtIRX10-L than for AtIRX10 indicating that IRX10-L is closer to the ancestral form of the protein. The expression of *PpGT47A* in *Physcomitrella* tissues undergoing primary cell wall formation contrasts with that of the *Arabidopsis IRX10* gene which is associated with tissues forming secondary cell walls. In vascular plants, the *IRX10* family appears to have undergone a sub- or neo-functionalisation resulting in at least a subset of the genes becoming specialised for secondary cell wall formation.

Analysis of the monosaccharide sugar composition of cell walls isolated from the *irx10 irx10-L* lines partially complemented with the *PpGT47A* gene does not show any significant changes in comparison to the *irx10 irx10-L* double mutant indicating that either changes are in a minor component of the cell wall and thus below the detection limit or that the PpGT47A function is not related to the cell wall composition. Although current evidence strongly indicates that IRX10 and related proteins from other plants function in glucuronoxylan biosynthesis, there have been previous reports suggesting involvement of the tobacco NpGUT1, another member of the IRX10 family of proteins, in rhamnogalacturonan II (RGII) biosynthesis, adding GlcA to one of the side chains of the RGII polysaccharide [48]. The decrease in the level of GlcA reported for the *Npgut1* tobacco mutant is greater than can be explained by a reduction in RGII levels alone arguing against NpGUT1 having a sole function in RGII synthesis. Interestingly, the *Npgut1* mutant also exhibits a decrease in xylose content, implying that xylan synthesis could also be affected. Furthermore, the tobacco NpGUT1 protein is able to perform the same enzymatic function as the *Arabidopsis* IRX10, restoring xylan levels to those of the wild-type when fused with the N-terminal 71 amino acid domain of the AtIRX10 protein and expressed in the *irx10 irx10-L* background [35]. Although this result supports an involvement of NpGUT1 in glucuronoxylan biosynthesis, the possibility that members of the IRX10 family, including PpGT47A, could function in the synthesis of RGII or an RGII-like polymer cannot currently be discounted.

### The role of PpGT47A in *physcomitrella*

A major advantage of using *Physcomitrella* is the possibility to make clean loss of function mutants by homologous recombination. This approach was used to create a knockout mutant for the *PpGT47A* gene. However, the knockout mutant had no obvious phenotypes under the conditions tested, nor did monosaccharide analysis of the gametophore cell wall reveal any significant changes in the mutant (Figure 6). We conclude from this that loss of *PpGT47A* function either does not affect the cell wall composition, or alternatively that any such effects are too subtle to be detected in our analysis. It should be noted that RGII and xylan are both known to be minor components of the bryophyte cell wall [9,10], and alterations in the composition of either or both polymers might therefore have escaped detection in our analysis of total cell wall material.

The absence of an obvious knockout phenotype could be due to several reasons. It is possible that the *PpGT47A* gene is functionally duplicated in *Physcomitrella*. Although this is unlikely since there are no close homologues of *PpGT47A* in the *Physcomitrella* genome [49], the possibility exists that another more distantly related GT can provide the same function as PpGT47A. Alternatively, it is possible that the modifications catalyzed by PpGT47A are important in *Physcomitrella* only under certain conditions, such as abiotic stress, but not under standard laboratory conditions. In any case, the fact that *PpGT47A* is strongly conserved and shows no evidence of being a pseudogene suggests that it encodes a functional protein, a conclusion that is reinforced by its ability to partially complement the *Arabidopsis irx10 irx10-L* double mutant.

## Conclusions

Genes encoding the enzymes required for synthesis of the GX backbone are highly conserved between *Physcomitrella*, *Arabidopsis* and *Populus* highlighting the importance of the polysaccharide throughout the plant kingdom*.* While the precise function of the *Physcomitrella GT47A* gene remains to be elucidated, its ability to partially complement the *Arabidopsis irx10 irx10-L* double mutant demonstrates that it encodes a GT with similar functional specificity to AtIRX10 and AtIRX10-L. Interestingly the absence of homologs of *IRX8* and *PARVUS* in the *Physcomitrella* genome indicates that the ability to synthesise the reducing end oligosaccharide of GX is an adaptation only found in higher plants. It is tempting to speculate that evolution of the GX biosynthesis machinery may have been a key step in the evolution of higher plants.

## Methods

### Bioinformatics

The *Physcomitrella patens* subsp *patens* v1.1 database and the Phytozome *Physcomitrella* database (http://www.phytozome.net/physcomitrella.php) were used to search for homologs using the *Arabidopsis* IRX10, IRX10-L, IRX9, IRX9-L, IRX14, IRX14-L, IRX8, PARVUS, GUX1 and GUX2 proteins. Sequences were aligned using Clustal-W software. Phylogenetic trees (Figure 2) were computed using the neighbour-joining method [46] as implemented in the Clustal-X software. The Phobius signal peptide and transmembrane prediction software was used for topological analysis (phobius.sbc.su.se) [44,47].

### Growth conditions

*Arabidopsis* was grown under long day conditions (16h light/8h dark) at a maximum irradiation of 150 μmolm^-2^s^-1^, 60% humidity with 22°C day and 18°C night temperatures. *Physcomitrella* gametophores were grown on BCD media (0.001 M MgSO_4_, 0.0189 mM KH_2_PO_4_, 0.01 M KNO_3_, 0.045 mM FeSO_4_, alternative trace element solution (TES) as described on http://moss.nibb.ac.jp/) and protonemata tissues were grown on MM media (BCD media with 5mM ammonium tartrate). Light, humidity, and temperature conditions employed were as for *Arabidopsis*.

### Overexpression and knock-out constructs

*PpGT47A* was PCR amplified from *Physcomitrella* DNA using forward primer (F): 5^′^-ggggacaagtttgtacaaaaaagcaggctgggagaattgggtgtttcg-3^′^ and reverse primer (R): 5^′^-ggggaccactttgtacaagaaagctgggtgtgttacaaatcattgcccc-3^′^), cloned into pDONR207 and then transferred into the pEarleyGate 100 destination vector [50] using the Gateway cloning system. The *PpGT47A* overexpression construct was introduced into the *Arabidopsis irx10 irx10-L*(+/-) background [36] using the floral dip method [51]. Transformed lines were screened using BASTA (Hoechst Schering AgrEvo GmbH, Germany) selection and *irx10 irx10-L* double mutants identified by PCR [35] allowing lines containing the transgene in a homozygous *irx10 irx10-L* background to be selected in the T2 generation. Wild-type and transformed mutant seed were sown and treated as previously described [35].

To generate the *PpGT47A* knock-out construct, the *NptII* gene was PCR amplified from the pMT164 vector, introducing an XmaI restriction site (F: 5^′^-aattcccggggagtcaaag-3^′^ and R: 5^′^-atggatcgatgttaacatgc-3^′^). *PpGT47A* was PCR amplified from *Physcomitrella* Gransden 2004 (F: 5^′^-ggacatagaagcatgatgc-3^′^ and R: 5^′^-ggaatacaacacgattcc-3^′^) and cloned into TOPO2.1 vector. The TOPO2.1 vector containing the *PpGT47A* gene was cut by XmaI and MfeI, the *NptII* cassette was cut with XmaI and EcoRI, and the two DNA fragments ligated using T4 DNA-ligase. The resulting construct was linearized by EcoRI before being transformed into Gransden 2004 wild-type protoplasts [52] using PEG mediated transformation as described previously [53]. Stable transformants were identified by selection on kanamycin-containing media for 2 weeks, followed by growth on selection free media for 2 weeks and then a further hygromycin selection for 2 weeks. Transformants were confirmed by PCRs spanning the recombination sites. Primers used for verification of the knockout construct were F1: tggtcaggagaatcatgc and F2: cggaagtaacagaatgagg, R1 ttgataactgtgggttacc and R2: caggtgacatgagactcg, and for the insert F: ttcgctcatgtgttgagc and R: aggcatcttcaacgatgg. All restriction enzymes used were FastDigest, Fermentas (St. Leon Rot, Germany).

### GUS construct

The GUS reporter gene was fused to the *PpGT47A* gene in the *Physcomitrella* genome using the following strategy. Two fragments, one homologous to the DNA sequence upstream and one to the region downstream of the *PpGT47A* stop codon, were amplified from endogenous *Physcomitrella* Gransden 2004 DNA by PCR. The PCR for the upstream fragment introduced a BamHI and a XbaI site at the 5^′^ end with the forward primer and the stop codon was disrupted with the reverse primer (F: 5^′^-ggatccttctttctagtgacaatcgg-3^′^ and R: 5^′^-cgcggatccacaaatcattgccccaagg-3^′^). The downstream fragment was amplified using F: 5^′^-ctttcttggaatactcacc-3 and R: 5^′^-ctggaacgcatctagacc-3^′^ primers. The fragments were cloned separately into the Topo2.1 vector. The cloned downstream fragment was excised with NotI and XbaI and ligated into a modified Topo vector carrying the GUS gene [54]. The upstream PCR fragment was cut with BamHI and introduced into Topo vector carrying the downstream fragment and the GUS gene. All restriction enzymes were FastDigest, Fermentas.

### GUS staining

The composition of the BCD media and the growth conditions in the light chamber were as previously described [45]. Clumps of subcultured protonema tissue were placed on BCD plates and grown for three weeks in continuous light at 25°C and then moved to short day conditions (8 hours light/16 hours dark at 15°C) and grown for three months. GUS staining was performed by incubating the moss tissue in X-gluc substrate solution as described by the Physcobase protocol (http://moss.nibb.ac.jp/). Stained tissue was analyzed with an Olympus SZX12 stereo microscope and images recorded using an Olympus XC30 camera.

### Phenotyping of *Ppgt47A* knockout lines

Small clumps of subcultured protonema tissue (approximately 2 mm in diameter) were used to inoculate standard BCD plates and plates containing different additions. The additions were 0.15 M glucose, 0.15 M fructose, 0.15M sucrose, 0.15 M mannitol or 5 mM ammonium tartrate. The plates were placed vertically in an incubator under three different conditions, high light (30 μmol/m^2^s), low light (6 μmol/m^2^s) or complete darkness. In addition, morphology studies were carried out on *Physcomitrella* grown on BCD plates containing the following conditions and concentrations of hormones. High light: 7 μM BAP, 1 μM ABA, 0.5 μM IAA and 10 μM GA3. Low light: 0.5 μM BAP, 0.5 μM ABA, 0.5 μM IAA and 10 μM GA3. All hormones were from Sigma Aldrich (Stockholm, Sweden). Plates were observed for two months.

### Sugar analysis

Basal stem regions from wild-type *Arabidopsis* plants measuring 30 mm in height or 6 weeks old, 9-week old mutant or complemented plants and 8-week old *Physcomitrella* gametophores grown on BCD media were used for monosaccharide analysis. Tissues were collected in 80% ethanol and stored at -80°C until being freeze dried (Modulyo, Edwards, West Sussex, UK). Dried material was ball milled in a beadmill (Retsch MM301, Haan, Germany) for 2×30s at 30 Hz. Alcohol insoluble residues (AIR) were obtained as previously described [39]. The AIR material was suspended in 0.1 M phosphate buffer, pH7 containing 0.01% sodium azide. Alpha-amylase (Roche, Indianapolis, USA) was added at a concentration of 1000U per 1g of cell wall material and the material was digested with gentle shaking for 24h at 37°C. The procedure was repeated once before the pellet was washed first with 0.1 M phosphate buffer pH 7, then with water and finally acetone. The material obtained was analysed using the TMS method [55-57].

### Tissue sections

Basal stem segments were collected, fixed in FAA (5% Acetic acid, 50% ethanol, 5% formadehyde in dH_2_O) and stored at 4°C until being sectioned using a vibratome (60 μm thickness) (Leica VT1000S, Germany), stained with 1:2 filtered safranin (1% in 50% ethanol): alcian blue (1% in H_2_0, 1% formalin and 0.15% glacial acetic acid), rinsed in H_2_O and mounted in 50% glycerol [58].

## Abbreviations

GT: Glycosyltransferase; AGP: Arabinogalactan protein; GX: Glucuronoxylan; IRX: Irregular xylem; CaMV: Cauliflower Mosaic Virus; GlcA: Glucuronic acid; GA3: Gibberellic acid; ABA: Abscisic acid; BAP: 6-benzylamino purine; IAA: Indole-3-acetic acid; AIR: Alcohol insoluble residues.

## Competing interests

The authors declare that they have no competing interests.

## Authors’ contributions

EH carried out the bioinformatics, complementation experiments, cell wall analysis, generated all constructs and wrote the majority of the paper. MU performed the moss transformations, analysis of knockout phenotypes and GUS histochemistry. HR was involved in discussions, bioinformatics, writing the manuscript and provided facilities. AM conceived and supervised the project, and was involved in writing the paper. All authors read and approved the final manuscript.

## Supplementary Material

Additional file 1 Figure S1. Physcomitrella patens wild-type colony and Ppgt47a knock out colony. The plants were grown for 6 weeks on BCD media supplemented with 5 mM ammonium tartrate. A. Wild-type. B. Ppgt47a. Click here for file
