# Supplementary figures and images for: Partial functional conservation of IRX10 homologs in physcomitrella patens and Arabidopsis thaliana indicates an evolutionary step contributing to vascular formation in land plants
Source: BMC Plant Biol. 2013 Jan 3;13:3. doi: 10.1186/1471-2229-13-3 (PMC3543728; doi:10.1186/1471-2229-13-3)

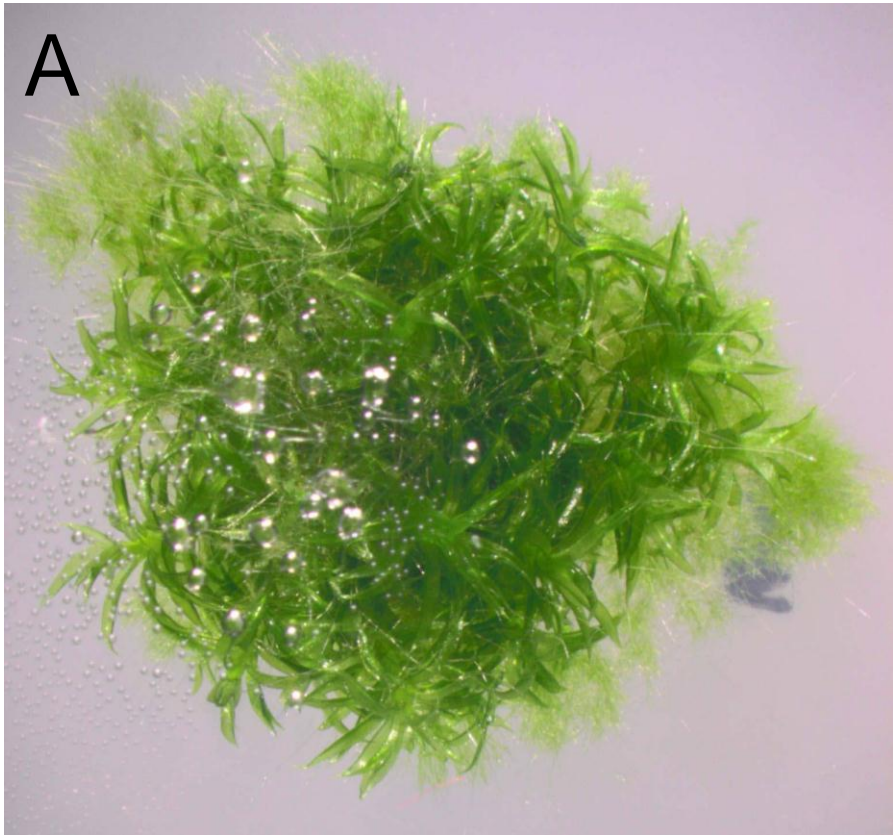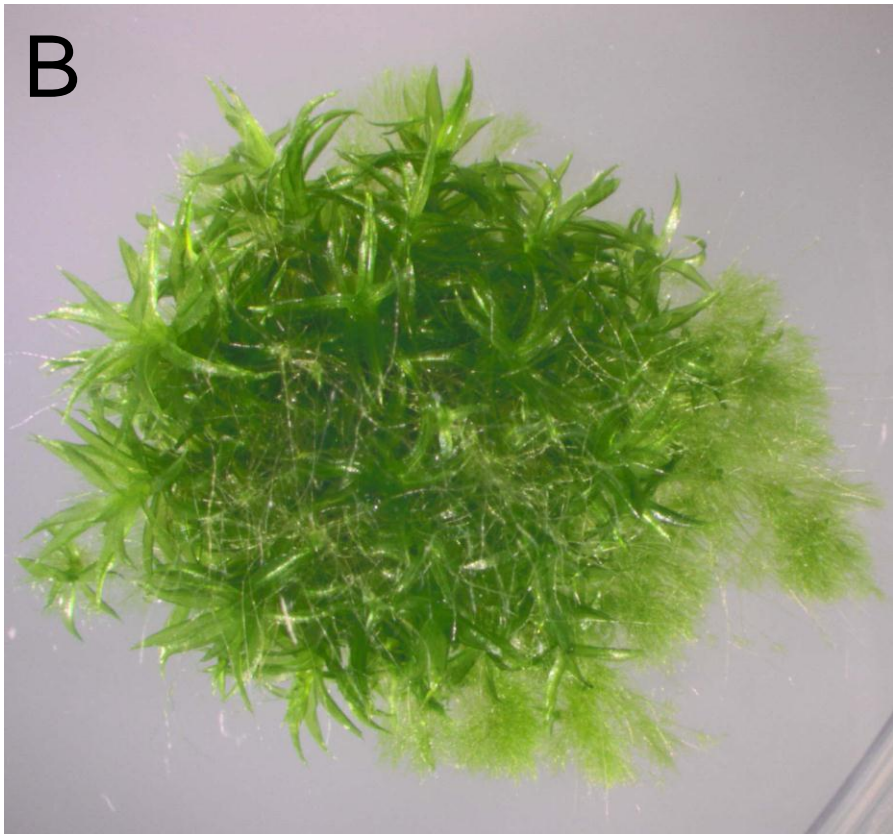

Supplement: Additional file 1 Figure S1. — Physcomitrella patens wild-type colony and Ppgt47a knock out colony. The plants were grown for 6 weeks on BCD media supplemented with 5 mM ammonium tartrate. A. Wild-type. B. Ppgt47a. [file 1471-2229-13-3-S1.pdf]
